# Long non-coding RNA FOXD3 antisense RNA 1 augments anti-estrogen resistance in breast cancer cells through the microRNA-363/ trefoil factor 1/ phosphatidylinositol 3-kinase/protein kinase B axis

**DOI:** 10.1080/21655979.2021.1962694

**Published:** 2021-08-23

**Authors:** Lili Ren, Huanhuan Zhou, Lei Lei, Yongjun Zhang, Hu Cai, Xiaojia Wang

**Affiliations:** a Department of Integration of Traditional Chinese and Western Medicine, the Cancer Hospital of the University of Chinese Academy of Sciences (Zhejiang Cancer Hospital), Institute of Basic Medicine and Cancer (IBMC), Chinese Academy of Sciences, Hangzhou, Zhejiang, P.R. China; b Department of Medical Oncology, the Cancer Hospital of the University of Chinese Academy of Sciences (Zhejiang Cancer Hospital), Institute of Basic Medicine and Cancer (IBMC), Chinese Academy of Sciences, Hangzhou, Zhejiang, P.R. China

**Keywords:** Breast cancer, anti-estrogen resistance, tamoxifen, lncRNA foxd3-as1, microRNA-363, trefoil factor 1 (tff1)

## Abstract

Long non-coding RNA (lncRNA) FOXD3 antisense RNA 1 (FOXD3-AS1) has been reported to participate in multiple processes that contribute toward the development of cancer. The present study aimed to explore the effect of lncRNA FOXD3-AS1 on anti-estrogen resistance in breast cancer (BC) cells. FOXD3-AS1 was found to be highly expressed in BC cell lines. Moreover, FOXD3-AS1 was highly expressed in estrogen receptor-negative (ER^−^) cells compared to the ER-positive (ER^+^) cells. FOXD3-AS1 overexpression in T47D and MCF-7 (ER^+^) cells enhanced the resistance of cells to tamoxifen (TMX), whereas FOX3-AS1 downregulation reduced the TMX resistance in MDA-MB-231 (ER^−^) cells. Similar results were reproduced *in vivo* that FOXD3-AS1 inhibition reduced the growth of xenograft tumors formed by MDA-MB-231 cells following TMX treatment whereas FOXD3-AS1 overexpression in T47D cells facilitated tumor growth. The bioinformatic analysis and luciferase assays indicated that FOXD3-AS1 sponged microRNA-363 (miR-363) to restore expression of trefoil factor 1 (TFF1) mRNA. Overexpression of miR-363 reduced T47D cell proliferation induced by FOXD3-AS1, whereas overexpression of TFF1 restored growth of MDA-MB-231 cells reduced after FOXD3-AS1 silencing. The phosphorylation of phosphoinositide 3-kinase (PI3K)/protein kinase B (Akt) was increased by FOXD3-AS1 but attenuated by miR-363. Inhibition of PI3K/Akt blocked the role of FOXD3-AS1 and reduced the TMX resistance in T47D and MCF-7 cells. Taken together, the present study suggested that FOXD3-AS1 sponges miR-363 to upregulate TFF1 expression, leading to PI3K/Akt signaling activation and anti-estrogen resistance in BC cells.

## Graphical Abstract

**Figure:**
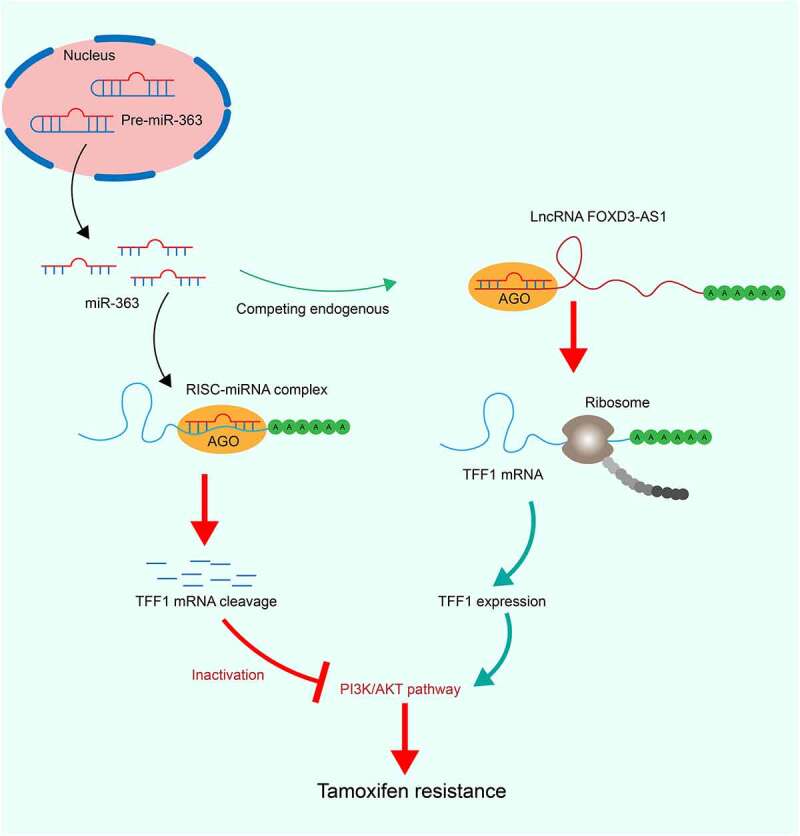

## Introduction

Breast cancer (BC), a heterogeneous disease comprising various clinical symptoms and significant tumor characteristics [1], is the most common malignancy among women worldwide [2]. The mortality rate for BC has declined over the course of the last few decades, and an early detection is crucial for a favorable outcome [3]. Approximately 75% of diagnosed cases of BC present with estrogen receptors (ERs), and these cases are termed ER-positive (ER^+^) BC; adjuvant endocrine therapies, such as treatment with tamoxifen (TMX) and aromatase inhibitors (AIs), have shown great potential in terms of reducing the risk of BC recurrence and increasing the survival rate [4,5]. In addition, TMX has also been observe to have treating effect on ER^−^ cells [6–8]. However, numerous patients will develop acquired resistance to anti-estrogen therapy following initial treatment, and ~50% of patients with advanced ER^+^ BC do not respond to TMX or AIs in the first-line treatment [9]. The mechanism of action underlying the acquired resistance response has yet to be elucidated, and therefore the identification of other molecular players that participate in the development of acquired resistance to adjuvant endocrine therapy is urgently required.

Estrogen-responsive BC cells typically undergo a range of basal and estrogen-mediated transcriptional events, including those that trigger coding and non-coding sense and antisense transcripts, which results in the transcription of multiple transcription units (predominantly genes) [10]. Gene intervention, holding great potential with respect to anti-estrogen therapy resistance, has attracted wide interest. Long non-coding RNAs (lncRNAs) are a class of RNA molecules >200 nucleotides in length that participate in a wide range of biological and pathological processes, including the development of BC and the emerging field of endocrine therapy resistance that has been identified [11]. For example, lincRNA-regulator of reprogramming [6] and lncRNA H19 [12] have been documented to fulfill key functions in the proliferation and survival of ER^+^ BC cells. LncRNA FOXD3 antisense RNA 1 (FOXD3-AS1) has been recently validated as a tumor promoter in several human cancer types, including colon adenocarcinoma [13] and thyroid cancer [14]. Interestingly, FOXD3-AS1 has been linked to clinical progression, regulating BC cell migration and invasion [15], although the underlying mechanism of has yet to be elucidated. Importantly, it is well established that a lncRNA may act as microRNA (miRNA) ‘sponges’ that compete for the mRNAs with shared miRNA-response elements at the binding sites, termed competing endogenous RNA (ceRNA) [16]. This ceRNA network has been validated in multiple human diseases, including in BC [17].

With the aid of bioinformatic analysis, FOXD3-AS1 was predicted to regulate expression of trefoil factor 1 (TFF1) mRNA through binding to miR-363. TFF1 has been indicated as one of the key genes associating with increased endocrine resistance and poor clinical outcome of BC [18,19]. We therefore, hypothesized that FOXD3-AS1 may restore the expression of TFF1 through sequestering its inhibitory miR-363. The goal of the present study was to validate the interactions between FOXD3-AS1, miR-363 and TFF1 mRNA and their functions in TMX resistance in BC both *in vivo* and *in vitro*.

## Materials and methods

### Antibodies, reagents, vectors, miRNAs and small interfering RNAs (siRNAs)

Primary antibodies against TFF1 (1:2,000 dilution; cat. no. ab92377, Abcam), Ki-67 (1:5,000; cat. no. sc23900, Santa Cruz Biotechnology, Inc.), glyceraldehyde-3-phosphate dehydrogenase (GAPDH, 1:5,000; cat. no. ab8245, Abcam), phosphoinositide 3-kinase (PI3K) (1:5,000; cat. no. ab151549, Abcam), Tyr^607^-phosphorylated PI3K (p-PI3K^Y607^) (1:5,000; cat. no. ab182651, Abcam), protein kinase B-1 (Akt1) (1:5,000; cat. no. ab235958, Abcam), secondary goat anti-rabbit antibody immunoglobulin G (IgG) (1:10,000; cat. no. ab6721, Abcam) and Ser^473^-phosphorylated Akt (p-Akt1^S473^) (1:5,000; cat. no. ab81283, Abcam) were used. The sequences of RNA overexpressing/interfering vectors were identified. The miR-363 mimic, mimic mock, siRNAs targeting FOXD3-AS1 and siRNA scramble, FOXD3-AS1- and TFF1-overexpressing vectors and empty vectors were all purchased from Guangzhou RiboBio Co., Ltd.

### Cell culture

The BC cell lines (MDA-MB-468, MDA-MB-453, SUM1315, MDA-MB-231, T47D and MCF7), and a human mammary epithelial cell line (MCF10A) were procured from ATCC (Manassas, VA, USA).The MDA-MB-468, MDA-MB-453 and SUM1315 cells were cultured in Dulbecco’s modified Eagle’s medium (DMEM)/F12 supplemented with 10% fetal bovine serum (FBS) (Thermo Fisher Scientific, Inc.), whereas the MDA-MB-231 cells were cultured in 10% FBS-supplemented Leibovitz’s L-15 medium (Thermo Fisher Scientific, Inc.). The T47D and MCF7 cells were also cultured in 10% FBS/DMEM. Cells were cultured at 37°C in an atmosphere lacking CO_2_. The aforementioned miRNA mimic or RNA overexpressing/interfering vectors were transfected into these cell lines using FuGENE® HD transfection reagent (Promega Corporation) according to the manufacturer’s protocol.

### Reverse-transcription quantitative polymerase chain reaction (RT-qPCR)

Total RNA from cells was extracted using an miRVana PARIS assay kit (Thermo Fisher Scientific, Inc.). Expression levels of lncRNAs and mRNAs were determined by qPCR using a GoTaq qPCR Master Mix kit (Promega Corporation), whereas the expression level of mature miR-363 was determined using a TaqMan RT-qPCR kit (Promega Corporation) with U6 as an internal reference. All procedures were performed according to the manufacturer’s instructions. The sequences of the primers are shown in Table 1.

**Table 1.** Primer sequences for RT-qPCR

Gene	Primer sequence (5ʹ-3ʹ)
FOXD3-AS1	F: AGGCCAACCAGAAGACC
	R: GAAGAGAGTCGCGCAC
miR-363	F: GGGTTGTGCTACAGATGATAGAG
	R: AGACGCCTCCTTTGTGTTAAT
TFF1	F: AGTACCCGTAGGCACAAC
	R: CGTGCTAGGACCGACTTCCTC
GAPDH	F: CCACCCCTATCCTGAAC
	R: CCCTGACTCTTATC
U6	F: TGAGGAGGAACAAGAAGATG
	R: ATCCAGACTCTGACCTTTT

Note: RT-qPCR, reverse transcription quantitative polymerase chain reaction; FOXD3-AS1, FOXD3 antisense RNA 1; miR: microRNA, TFF1, trefoil factor 1, glyceraldehyde-3-phosphate dehydrogenase; F: forward; R: reverse.

### Cell proliferation assays

Proliferation of cells was determined using 3-(4, 5-dimethylthiazol-2-yl)-2, 5-diphenyltetrazolium bromide (MTT), colony formation and 5-ethynyl-2ʹ-deoxyuridine (EdU) labeling assays. For MTT assay, at 24 h after transfection, cells were sorted into 96-well plates at a density of 5 × 10^3^ cells per well and subsequently incubated with 10 μl MTT solution (0.5 mg/ml; Millipore Sigma) at 37°C for 4 h. The culture solution was then removed, and the sedimented formazan was dissolved in 150 μl dimethyl sulfoxide solution. The optical density at 570 nm was determined using a microplate reader (Bio-Rad Laboratories, Inc.). As far as the colony formation assay was concerned, at 24 h after transfection cells were sorted into 6-well plates at a density of 500 cells per well. After 3 weeks, the colonies formed by cancer cells were washed in phosphate-buffered saline (PBS), fixed in 10% formalin for 15 min, and subsequently stained with hematoxylin. The number of colonies was then calculated. The EdU labeling assay was conducted using a Cell-Light Appolo488 EdU assay kit (Guangzhou RiboBio Co., Ltd.) according to the manufacturer’s instructions. The EdU-positive cells were observed under a fluorescence microscope, and the EdU-positive rate was calculated as follows: Rate = (EdU-labeled cells/Hoechst-stained cells) x100%.

### Subcellular localization of FOXD3-AS1

Fluorescence *in situ* hybridization (FISH) was performed to determine the subcellular localization of FOXD3-AS1. In brief, the fluorescence-labeled FOXD3-AS1 probes used for these experiments were designed by Guangzhou RiboBio Co., Ltd., and a FISH kit, also provided by Guangzhou RiboBio Co., Ltd., was used to detect the probe signals according to the manufacturer’s instructions. Nuclei of the MB-MDA-231 or T47D cells were stained by 4ʹ,6-diamidino-2-phenylindole (DAPI). Images of stained cells were then captured under a confocal microscope (Olympus Corporation).

### Xenograft tumors in nude mice

Stable overexpression of FOXD3-AS1 was induced in T47D cells, whereas FOXD3-AS1 expression was silenced in MDA-MB-231 cells. The respective cells were resuspended, harvested and mixed with Matrigel™ BD (Becton, Dickinson and Company). Subsequently, each BALB/c mouse was injected with 1 × 10^6^ cells through the mammary fat pad. When the tumor volume reached 100 mm^3^, the mice were treated with TMX (5 mg/pellet). After a further 8 weeks, the mice were euthanized by administration of an overdose of pentobarbital (120 mg/kg), and the volume and mass of the tumors were then measured. The animal procedures were approved by the Clinical Ethical Committee of Zhejiang Cancer Hospital and performed in line with the ethical guidelines for the study of experimental pain in conscious animals (National Institutes of Health, Bethesda, Maryland, USA).

### Immunohistochemistry

Tissue sections from xenograft tumors were baked at 60°C for 30 min, dewaxed in xylene for 5 min in triplicate, and then successively dehydrated in 100, 95 and 70% ethanol (three times for each concentration). Tissue sections were further treated with 3% H_2_O_2_ to block the endogenous peroxidase activity, and subsequently the sections were sealed with sheep serum for 1 h. The tissue sections were then incubated with diluted anti-Ki67 antibody (1:200) at 4°C overnight, followed by incubation with the secondary antibody at 37°C for 2 h. The tissues were then treated with diaminobenzidine. For each treatment group, 6 samples were randomly observed under a microscope at x200 and x400 magnifications with 5 fields included.

### Dual luciferase reporter gene assay

The binding interactions between miR-363 and FOXD3-AS, and between miR-363 and TFF1 3ʹuntranslated region (3ʹUTR), were first predicted using TargetScan (http://www.targetscan.org/vert_72/), prior to testing with dual luciferase reporter gene assay. The pMIR-REPORT^TM^ luciferase reporter vectors containing the FOXD3-AS1 wild-type (WT) and mutant-type (MT) binding sequences (Figure 3b), as well as the vectors containing TFF1 3ʹUTR WT and MT sequences (Figure 3d), were co-transfected with miR-363 mimic or NC mimic into 293 T cells for 36 h. The luciferase activity was detected using a dual-luciferase reporter gene system (Promega Corporation) according to the manufacturer’s instructions.

### Western blot analysis

Cells were lysed in proteinase inhibitor (Roche Molecular Diagnostics)-supplemented protein lysis buffer to collect total protein. The protein (50 μg) were separated using 8–12% sodium dodecyl sulfate-polyacrylamide gel electrophoresis and transferred onto polyvinylidene fluoride membranes (Merck KGaA). The membranes were incubated with primary antibodies at 4°C overnight and then with HRP-conjugated secondary antibody at 37°C for 2 h. The protein bands were observed using enhanced chemiluminescence reagent (Thermo Fisher Scientific). Protein level relative to GAPDH was examined using the Image J software.

### Statistical analysis

Data were analyzed using Statistical Product and Service Solutions (SPSS, version 21.0; IBM Corp.). Kolmogorov-Smirnov tests were performed to ensure that the data were normally distributed. The results obtained from at least three independent experiments were presented as the mean ± standard deviation (SD). Differences between every two groups were evaluated using the *t*-test, whereas differences among multiple groups were compared using one-way or two-way analysis of variance(ANOVA). Tukey’s multiple comparisons test was used for the post hoc test. P-values were obtained from two-tailed tests, and P < 0.05 was considered to indicate a statistically significant difference.

## Results

### Brief introduction

FOXD3-AS1 was predicted to serve as a ceRNA to restore the expression of TFF1 through sequestering its inhibitory miR-363. This study was therefore performed to validate the interactions between FOXD3-AS1, miR-363 and TFF1 mRNA and their functions in TMX resistance in BC. Luciferase reporter assays were performed to validate the binding relationships between FOXD3-AS1 and miR-363 and between miR-363 and TFF1 mRNA. Gain- and loss-of-function studies of FOXD3-AS1, miR-363 and TFF1 were performed in BC cells for both *in vitro* and *in vivo* experiments. A PI3K/Akt-specific antagonist PI3K-IN-1 was introduced in cells to validate the involvement of this signaling in the FOXD3-AS1-mediated events.

### Silencing of FOXD3-AS1 increases the sensitivity of BC cells to TMX

**Figure 1.**
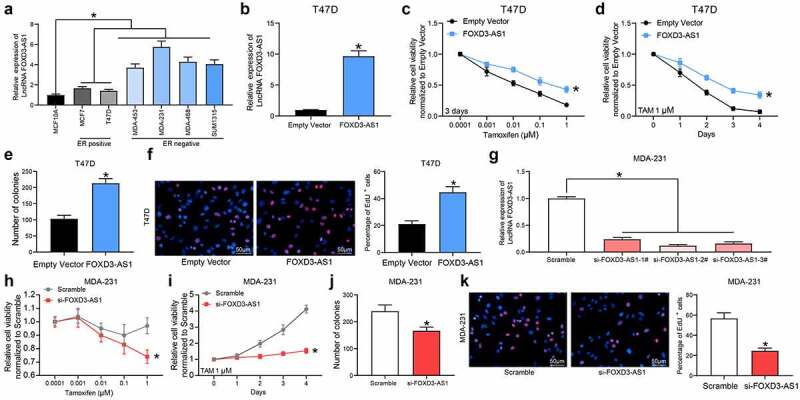
Silencing of FOXD3-AS1 increases the sensitivity of BC cells to TMX. A, expression of FOXD3-AS1 in BC cell lines and normal MCF10A cells, and in ER+ and ER- cell lines was determined using RT-qPCR. B, FOXD3-AS1 overexpressing vector and the corresponding empty vector were transfected into T47D (ER+) cells, and then FOXD3-AS1 expression in cells was determined using RT-qPCR. C-F, cells with overexpression of FOXD1-AS1 were treated with TMX, after which the cell viability and growth were identified by MTT (c-d), colony formation (e) and EdU labeling (f) assays. G, siRNAs targeting FOXD3-AS1 and the corresponding siRNA scramble were transfected into MDA-MB-231 cells, after which FOXD3-AS1 expression in cells was determined using RT-qPCR. H-K, cells with silencing of FOXD1-AS1 were treated with TMX, and then the cell viability and growth were identified by MTT (h-i), colony formation (j) and EdU labeling (k) assays. Repetition = 3. Data were exhibited as mean ± SD. In panels B, E, F, J and K, data were analyzed using unpaired *t* test, while data in panels A and G were analyzed using one-way ANOVA and data in panels C, D, H and I were analyzed using two-way ANOVA. Tukey’s multiple comparison test was used for post hoc test after ANOVA; *, *p* < 0.05

First, the expression of FOXD3-AS1 in BC cell lines and in normal MCF10A cells was examined. It was found that FOXD3-AS1 was upregulated in all BC cell lines compared with the MCF10A cells. Of note, the FOXD3-AS1 expression was increased in the ER-negative (ER^−^) cell lines (MDA-MB-453, MDA-MB-468, MDA-MB-231 and SUM1315) compared with the ER^+^ cell lines (MCF-7 and T47D) (Figure 1a), indicating that FOXD3-AS1 expression might be associated with resistance to endocrine therapies. Subsequently, FOXD3-AS1-overexpressing vector was introduced into T47D and MCF7 (ER^+^) cells (Figure 1b) (Supplementary Fig. S1A). Following TMX treatment, overexpression of FOXD3-AS1 was shown to lead to an increase in cell viability (i.e., increased TMX resistance) (Figure 1c and d) (Supplementary Fig. S1B-C). Colony formation assay showed that the number of cell colonies formed by T47D and MCF7 cells was increased following FOXD3-AS1 overexpression (Figure 1e) (Supplementary Fig. S1D). Moreover, the EdU labeling assay showed that the number of cells in S phase was increased when FOXD3-AS1 was overexpressed after TMX treatment (figure 1f) (Supplementary Fig. S1E). In a separate series of experiments, silencing of FOXD3-AS1 was introduced in MDA-MB-231 (ER^−^) cells (Figure 1g). This demonstrated that the cell viability (Figure 1h and i), the colony formation ability (Figure 1j), and the number of cells in S phase (Figure 1k) of MDA-MB-231 cells were all decreased following FOXD3-AS1 silencing.

### Silencing of FOXD3-AS1 enhances the sensitivity of BC cells to TMX in vivo

**Figure 2.**
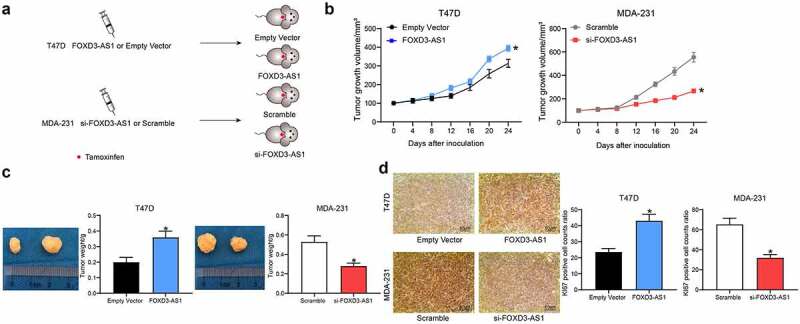
Silencing of FOXD3-AS1 enhances the sensitivity of BC cells to TMX *in vivo*. A, each nude mouse was implanted with 1 × 10^6^231-si-FOXD3-AS1 cells or T47D-FOXD-AS1 cells or the corresponding control cells through the mammary fat pads. When the tumor size reached about 100 mm^3^, the mice were given TMX treatment. B, the volume of tumor in each group of mice was determined every 4 d. C, the weight of tumor in each group of mice was determined at the experimental endpoint. D, Ki-67 expression in each group of mouse tumors was determined by immunohistochemistry. In each group, n = 6. Repetition = 3. Data were exhibited as mean ± SD. Data in panel B were analyzed using two-way ANOVA, while data in panels C and D were analyzed using one-way ANOVA, and Tukey’s multiple comparison test was used for post hoc test. *, *p* < 0.05

The above-mentioned findings prompted us to investigate the role of FOXD3-AS1 silencing in anti-estrogen sensitivity *in vivo*. MDA-MB-231 cells with silenced FOXD3-AS1 (defined hereafter as ‘231-si-FOXD3-AS1 cells’) or T47D cells with overexpressed FOXD-AS1 (similarly defined as ‘T47D-FOXD-AS1 cells’) were implanted into BALB/c nude mice (Figure 2a), and their effects on the mice were monitored upon treatment of the mice with TMX. As shown in Figure 2b and 2c, the growth rate of xenograft tumors in mice implanted with 231-si-FOXD3-AS1 cells was significantly decreased following TMX treatment (scramble tumors compared with si-FOXD3-AS1 tumors: 600 vs. 220 mm^3^), whereas the opposite trend was revealed in mice injected with T47D-FOXD-AS1 cells (tumor sizes of empty vector vs. FOXD3-AS1 mice: 300 vs. 400 mm^3^). In addition, the expression of the proliferation marker Ki-67 in xenograft tumor tissues was examined by immunohistochemistry analysis. It was observed that silencing of FOXD3-AS1 reduced the number of Ki67-positive cells in tumors, whereas, accordingly, overexpression of FOXD3-AS1 led to an increase in the number of Ki67-positive cells in xenograft tumors (Figure 2d).

### FOXD3-AS1 upregulates TFF1 expression through sponging miR-363

**Figure 3.**
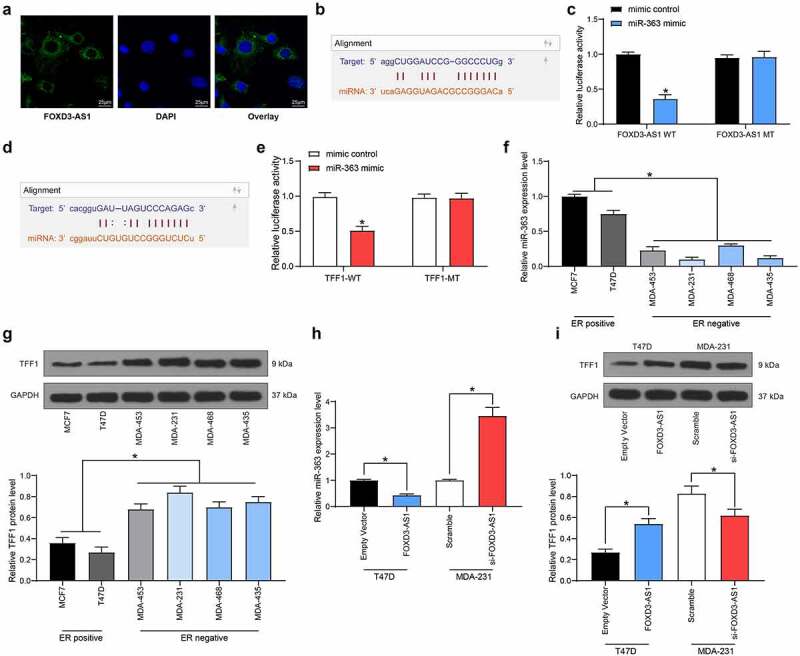
FOXD3-AS1 up-regulates TFF1 expression through sponging miR-363. A, the sub-cellular location of FOXD3-AS1 was determined by FISH assay. B-C, the binding relationship between FOXD3-AS1 and miR-363 was predicted on TargetScan (http://www.targetscan.org/vert_72/) (b) and validated by a dual luciferase reporter gene assay (c). D-E, the binding relationship between miR-363 and TFFI mRNA was predicted on TargetScan (d) and validated by a dual luciferase reporter gene assay (e). F-G, expression of miR-393 (f) and TFF1 mRNA (g) in ER+ and ER- cells was determined by RT-qPCR. H, miR-363 expression in MDA-MB-231 and T47D cells after FOXD3-AS1 interference was determined by RT-qPCR. I, protein level of TFF1 in MDA-MB-231 and T47D cells after FOXD3-AS1 interference was detected by western blot analysis. Repetition = 3. Data were exhibited as mean ± SD. Data in panels B and E were analyzed using two-way ANOVA while data in panels F, G, H and I were analyzed using one-way ANOVA, and Tukey’s multiple comparison test was used for post hoc test. *, *p* < 0.05

To further identify the potential mechanisms involved in FOXD3-AS1-mediated resistance in anti-estrogen treatment, the subcellular localization of FOXD3-AS1 was first examined. The FISH assay suggested that FOXD3-AS1 was mainly sublocalized in the cytoplasm (Figure 3a). Subsequently, miR-363 was identified as a target of FOXD3-AS1 according to the online prediction of the TargetScan system (Figure 3b). The FOXD3-AS1 WT vector containing the binding sites between the FOXD3-AS1 3ʹ-UTR and miR-363, and the corresponding MT vector was inserted into the downstream luciferase open-reading frame, with the result that the luciferase activity was decreased in 293T cells co-transfected with FOXD3-AS1 WT vector and miR-363 mimic (Figure 3c). Likewise, TFF1 was also identified as a target gene of miR-363 with TargetScan (Figure 3d), and this prediction was validated using the dual luciferase reporter gene assay (Figure 3e). Having the opposite effect to that of FOXD3-AS1 expression, miR-363 expression was significantly higher in ER^+^ cells compared with that in ER^−^ cells (figure 3f), whereas TFF1 expression was consistent with FOXD3-AS1 expression (Figure 3g). Moreover, MDA-MB-231 cells with overexpressed FOXD3-AS1 exhibited decreased expression levels of miR-363, whereas those of TFF1 were increased, and accordingly, T47D cells with silenced FOXD3-AS1 revealed the opposite trends (Figure 3h-i).

### Overexpression of miR-363 enhances the sensitivity of BC cells to TMX

**Figure 4.**
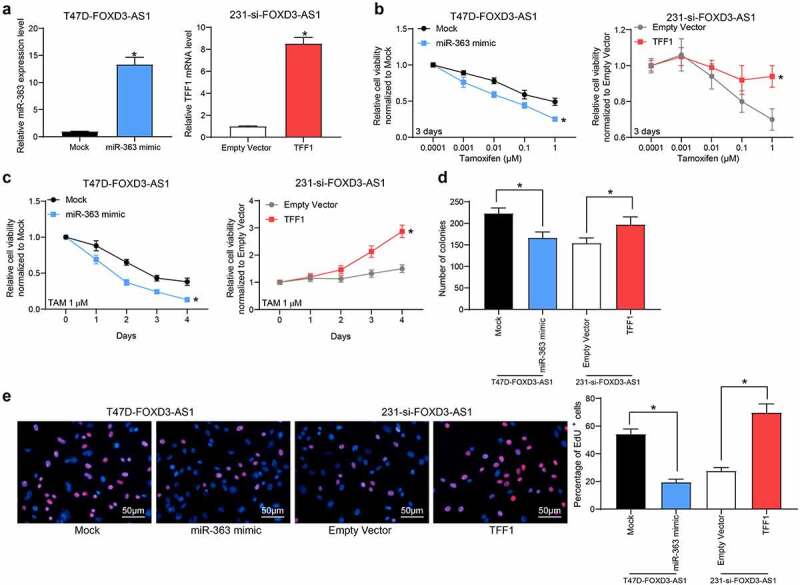
Overexpression of miR-363 enhances the sensitivity of BC cells to TMX. Overexpression of miR-363 was further introduced in T47D-FOXD3-AS1 cells, while overexpression of TFF1 was introduced in 231-si-FOXD3-AS1 cells. A, the expression of miR-363 and TFF1 mRNA in cells was determined by RT-qPCR. B-E, cell viability and growth were detected by MTT (b-c), colony formation (d) and EdU labeling (e) assays. Repetition = 3. Data were exhibited as mean ± SD. In panels A, D and E, one-way ANOVA was used for data analysis, while data in panels B and C were analyzed using two-way ANOVA, and Tukey’s multiple comparison test was used for post hoc test after ANOVA. *, *p* < 0.05

In light of the above findings, artificial overexpression of miR-363 was subsequently introduced into T47D-FOXD3-AS1 or MCF7-FOXD3-AS1 cells, whereas overexpression of TFF1 was introduced into 231-si-FOXD3-AS1 cells (Figure 4a) (Supplementary Fig. S2A). These experiments revealed that the cell viability (Figure 4b-c) (Supplementary Fig. S2B-C), and the colony formation (Figure 4d) (Supplementary Fig. S2D) and DNA replicative abilities of T47D cells (Figure 4e) (Supplementary Fig. S2E) promoted by FOXD3-AS1 were partly reduced following miR-363 mimic transfection. By contrast, the cell viability, colony formation and DNA replication abilities of cells inhibited after FOXD3-AS1 silencing were restored by TFF1 overexpression (Figure 4b-e).

### FOXD3-AS1 promotes TMX-resistance of BC cells through the PI3K/Akt signaling pathway

**Figure 5.**
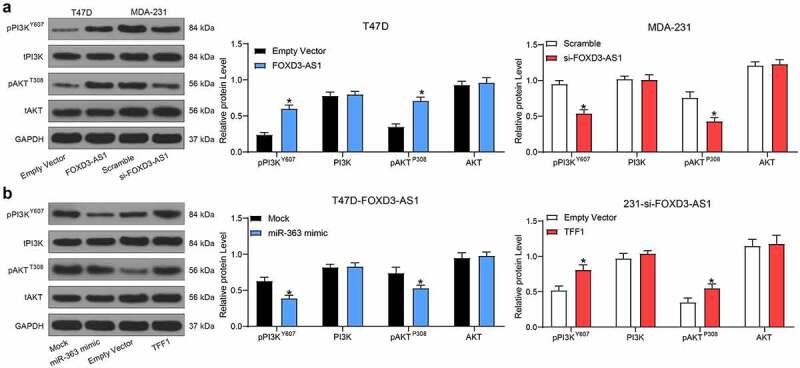
FOXD3-AS1 promotes TMX-resistance of BC cells through the PI3K/Akt signaling pathway. A, the protein level and phosphorylation of PI3K/Akt in T47D-FOXD3-AS1 and 231-si-FOXD3-AS1 cells were determined by western blot analysis. B, miR-363 mimic was transfected into T47D-FOXD3-AS1 cells and TFF1 overexpressing vector was administrated in 231-si-FOXD3-AS1 cells, and then the protein level and phosphorylation of PI3K/Akt signals were determined by western blot analysis. Repetition = 3. Data were exhibited as mean ± SD and analyzed using two-way ANOVA and Tukey’s multiple comparison test was used for post hoc test after ANOVA. *, *p* < 0.05

To investigate which downstream molecules may have been influenced by the FOXD3-AS1/miR-363/TFF1 axis, the protein level and extent of phosphorylation of PI3K/Akt were measured by western blot analysis. The results suggested that the phosphorylation of PI3K/Akt was at a higher level in T47D-FOXD3-AS1 cells compared with that in T47D-EV cells. By contrast, the extent of phosphorylation of PI3K/Akt was significantly inhibited in 231-si-FOXD3-AS1 cells (Figure 5a) (Supplementary Fig. S1F). In addition, further transfection of miR-363 mimic in T47D-FOXD3-AS1 cells led to a significant decline in the phosphorylation of PI3K/Akt, although transfection of TFF1-overexpressing vector in 231-si-FOXD3-AS1 cells led to the opposite trend (Figure 5b) (Supplementary Fig. S2F).

### Inhibition of the PI3K/Akt signaling pathway enhances the sensitivity of BC cells to TMX

**Figure 6.**
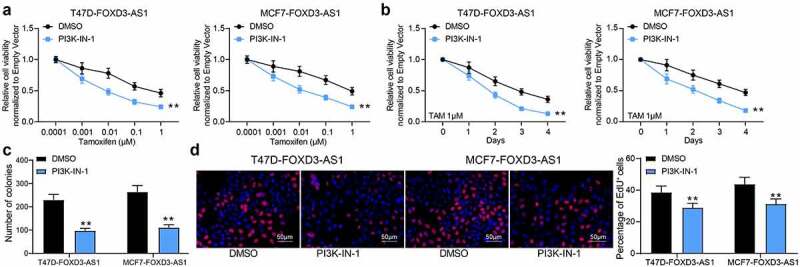
Inhibition of the PI3K/Akt signaling pathway enhances the sensitivity of BC cells to TMX. A-D, cell viability and growth were detected by MTT (a-b), colony formation (c) and EdU labeling (d) assays. Repetition = 3. Data were exhibited as the mean ± SD. In all panels, data were analyzed by two-way ANOVA followed by Tukey’s multiple comparison test. *, *p* < 0.05

To validate the involvement of the PI3K/Akt signaling in the TMX resistance in BC cells, the T47D-FOXD3-AS1 and MCF7-FOXD3-AS1 cells were treated with a PI3K/Akt-specific antagonist PI3K-IN-1, and cells treated with DMSO were set as the control. The cells were subjected to TMX treatment. Importantly, it was found that the viability of T47D and MCF7 cells increased by FOXD3-AS1 was blocked after PI3K-IN-1 treatment (Figure 6a-b). The number of cell colonies (Figure 6c) and cells in the S phase (Figure 6d) were also suppressed after PI3K-IN-1 treatment. These results indicated that the PI3K/Akt signaling activation is involved in the anti-sensitizing effects of FOXD3-AS1.

## Discussion

Estrogen signaling fulfills a crucial role in the regulation of growth and survival of estrogen-dependent tumors that account for the majority of cases of BC, signifying that endocrine therapy is a promising measure aimed at the treatment of ER^+^ BC cells [12]. However, resistance to endocrine therapy remains a major challenge in drug administration to patients with BC. FOXD3-AS1 has recently been identified to be expressed at aberrant levels in several human malignancies, including BC [15]. The present study demonstrated that FOXD3-AS1 may be associated with anti-estrogen therapy resistance of BC cells, with the FOXD3-AS1/miR-363/TFF1 ceRNA network and the PI3K/Akt signaling pathway possibly being involved in the process.

LncRNAs have been emergingly recognized to participate in BC progression by mediating resistance to endocrine therapy or chemotherapies [20,21]. As far as the regulation in anti-endocrine resistance, lncRNA in non-homologous end joining pathway 1, for instance, has been proposed to downregulate the protein level of ER and to attenuate the estrogen response, leading to increased anti-estrogen resistance [22]. Likewise, linc-regulator of reprogramming has been demonstrated to trigger the transition of ER^+^ BC cells from an estrogen-dependent to an estrogen-independent state, a key step in promoting endocrine therapy resistance [23]. FOXD3-AS1 is an oncogene that has recently been identified to be involved in several human malignancies, including cutaneous malignant melanoma [24] and glioma [25]. High expression of FOXD3-AS1 has also been observed in BC tissues and cells, which was associated with growth and metastasis of BC cells [15]. However, the role of this lncRNA in endocrine therapy resistance has not been investigated. In the present study, FOXD3-AS1 was first found to be highly expressed in ER^−^ cell lines compared with the ER^+^ ones. Importantly, overexpression of FOXD3-AS1 in T47D and MCF7 cells increased the resistance to death of cells following TMX treatment. Moreover, the growth rate of tumors in nude mice implanted with T47D-FOXD3-AS1 cells was accelerated. The tumors in these mice also exhibited increased levels of Ki67, a well-known proliferation marker that independently predicts cancer progression [26]. In agreement with this, in a study by Zeng *et al*., FOXD3-AS1 was found to augment the resistance of lung cancer cells to cisplatin [27]. Here, this study reported that FOXD3-AS1 might increase the resistance of BC cells to endocrine therapy.

The findings described above prompted us to further explore which molecules are involved in the underlying mechanism(s) associated with the FOXD3-AS1-mediated events. After identification of a cytoplasm-distribution of FOXD3-AS1 in BC cells, the bioinformatics analysis and luciferase assays collectively suggested that FOXD3 binds to miR-363 and is able to diminish its suppressive effects on TFF1 mRNA. Importantly, an inverse correlation between miR-363 and FOXD3-AS1, whereas a positive correlation between TFF1 and FOXD3-AS1 were identified in BC cell lines, whereas the opposite was observed with TFF1. miR-363 has been identified as a tumor inhibitor in a multitude of cancer types [28–30]. In patients with BC, miR-363 has also been reported to be associated with lower rates of recurrence [31]. As far as drug sensitivity is concerned, miR-363 has been found to inhibit chemoresistance of epithelial ovarian cancer cells to cisplatin [32,33]. In BC, miR-363 has been suggested to sensitize cisplatin-induced apoptosis in MDA-MB-231 cells [23]. Of more relevance to the present study, a recent report by Li *et al*. suggested that upregulation of miR-363 was associated with increased sensitivity of the TMX-resistant BC cells [34]. In the present study, miR-363 was identified as a sponge for FOXD3-AS1 and its upregulation increased the TMX-induced apoptosis in T47D cells that was initially reduced by FOXD3-AS1. TFF1 is a well-known biomarker in patients with BC [35,36]. Moreover, it has been demonstrated to be upregulated by estrogen in BC cells, and is associated with resistance to doxorubicin‑induced apoptosis [37]. The findings of the present study also disclosed that overexpression of TFF1 could promote the growth of 231-si-FOXD3-AS1 cells, indicating its similar role in promoting TMX-resistance in BC cells.

The PI3K/Akt signaling pathway is crucially important in tumorigenesis and key cellular processes, and its activation has been documented to lead to poor prognosis and resistance to chemotherapy of patients with BC [38]. More specifically, this signaling pathway has been suggested to exert critical functions in resistance to endocrine therapy in ER^+^ cells [39]. This hypothesis was strengthened by the discovery that an AKT antagonist, AZD5363, has been reported to re-sensitize the drug-resistance of cells to TMX when it synergistically acts with fulvestrant [40]. The present study revealed that the phosphorylation of PI3K/Akt was increased in T47D and MCF-7 cells overexpressing FOXD3-AS1 but was reduced in MDA-MB-231 cells with FOXD3-AS1 silencing. Importantly, treatment with PI3K-IN-1, a PI3K/Akt-specific antagonist, was found to significantly reduce TMX resistance induced by FOXD3-AS1 in T47D and MCF-7, validating that the PI3K/Akt activation was involved in the anti-sensitizing effect of FOXD3-AS1 on TMX treatment.

## Conclusion

In conclusion, the present study identified a novel FOXD-AS1/miR-363/TFF1 ceRNA network involved in the resistance of BC cells to TMX, in which the PI3K/Akt signaling pathway is activated. Silencing of FOXD-AS1 may serve as a novel therapeutic intervention option in terms of endocrine therapy targeting ER^+^ BC treatment. However, the mechanism by which TFF1 mediates the PI3K/Akt signaling pathway has not been elucidated yet, and further studies in the near future will focus on this issue in order to better understand the underlying molecular mechanisms involved in anti-estrogen resistance in BC cells.

## Supplementary Material

Supplemental Material

## Data Availability

The datasets used and/or analyzed during the current study are available from the corresponding author on reasonable request.

## Highlights

- FOXD3-AS1 is highly expressed in ER-negative BC cells versus ER-positive cells.
- Silencing of FOXD3-AS1 promotes the sensitivity of BC cells to TMX.
- Silencing of FOXD3-AS1 inhibits growth of BC cells *in vivo* after TMX treatment.
- FOXD3-AS1 promotes TFF1 expression through sponging miR-363.
- FOXD3-AS1 may enhance TMX-resistance of BC cells through the PI3K/Akt axis.
