# Supplementary material for: Long non-coding RNA FOXD3 antisense RNA 1 augments anti-estrogen resistance in breast cancer cells through the microRNA-363/ trefoil factor 1/ phosphatidylinositol 3-kinase/protein kinase B axis
Source: Bioengineered. 2021 Aug 23;12(1):5266–78. doi: 10.1080/21655979.2021.1962694 (PMC8806484; doi:10.1080/21655979.2021.1962694)
Supplement: Supplemental Material [file KBIE_A_1962694_SM9633.zip › Supplementary Figures.docx]

**Supplementary Fig. S1**


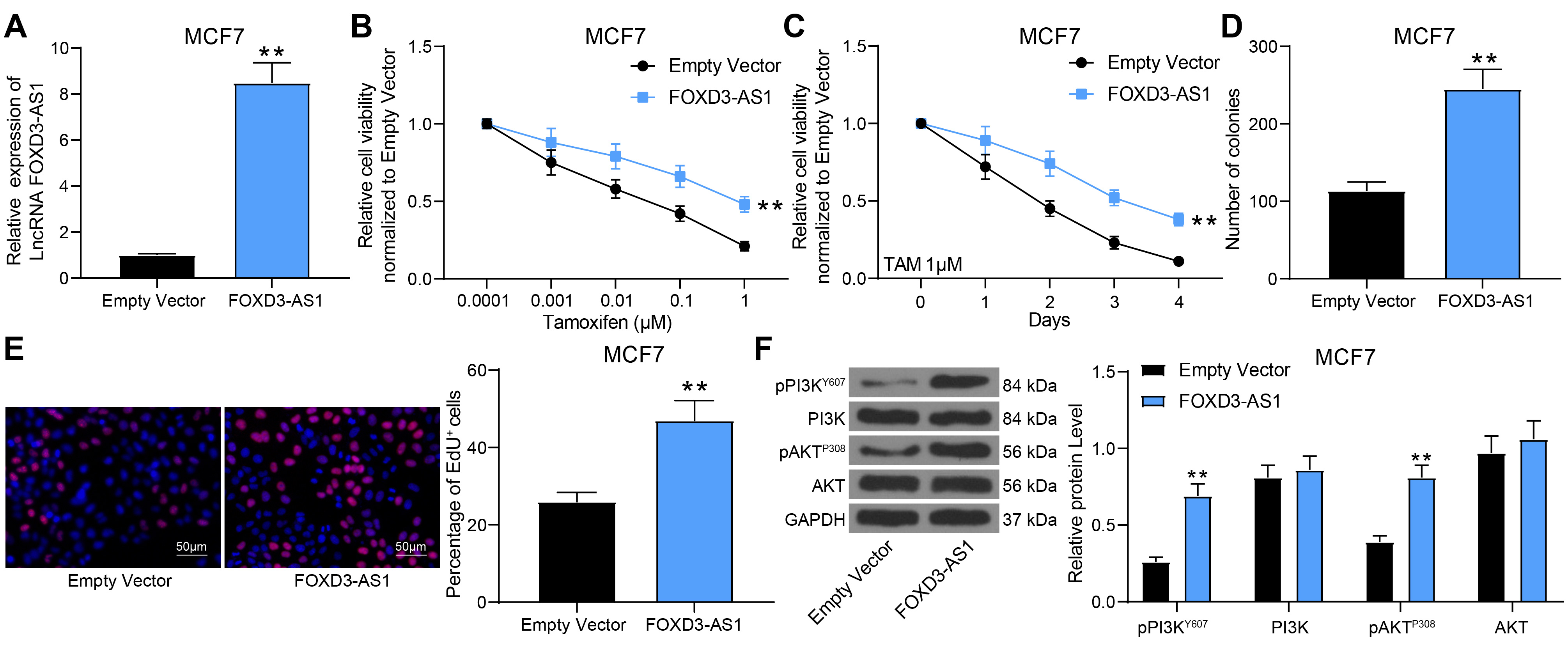


**Supplementary Fig. S1** Overexpression of FOXD3-AS1 increased the resistance of MCF-7 cells to TAM. A, the expression of FOXD3-AS1 in cells was determined by RT‑qPCR. B‑E, cell viability and growth were detected by MTT (B‑C), colony formation (D) and EdU labeling (E) assays. F, the protein level and phosphorylation of PI3K/Akt in MCF7 cells overexpressing FOXD3-AS1 were determined by western blot analysis. Repetition = 3. Data were exhibited as mean ± SD. In panels A, D and E, one‑way ANOVA was used for data analysis, while data in panels B, C and F were analyzed using two‑way ANOVA. Tukey’s multiple comparison test was used for post hoc test after ANOVA. *, p < 0.05.

**Supplementary Fig. S2**

**
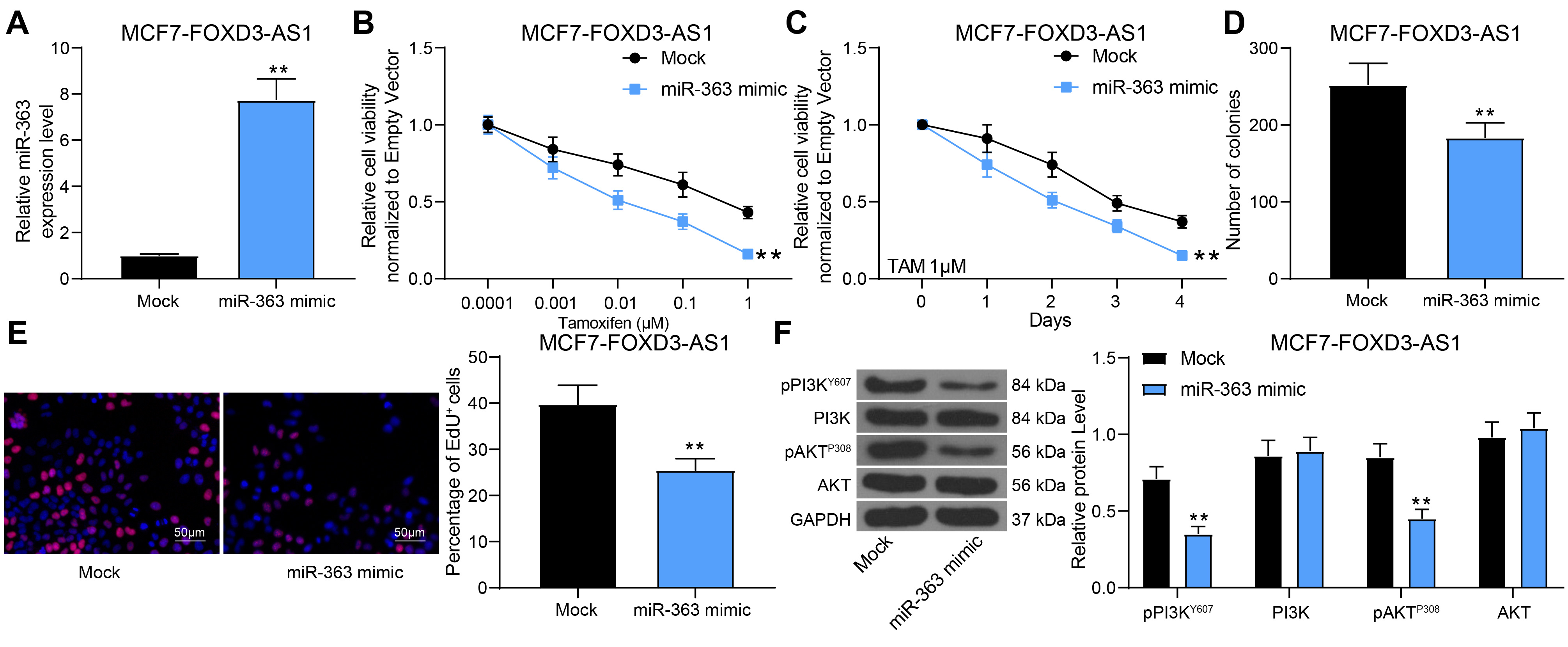
**

**Supplementary Fig. S2** Overexpression of miR‑363 enhances the sensitivity of MCF-7cells to TMX. A, the expression of miR-363 in MCF7-FOSD3-AS1 cells was determined by RT‑qPCR. B‑E, cell viability and growth were detected by MTT (B‑C), colony formation (D) and EdU labeling (E) assays. F, the protein level and phosphorylation of PI3K/Akt in MCF7-FOSD3-AS1 cells after miR-363 mimic transfection were determined by western blot analysis. Repetition = 3. Data were exhibited as mean ± SD. In panels A, D and E, one‑way ANOVA was used for data analysis, while data in panels B, C and F were analyzed using two‑way ANOVA. Tukey’s multiple comparison test was used for post hoc test after ANOVA. *, *p* < 0.05.
